# FTO modifies the m6A level of MALAT and promotes bladder cancer progression

**DOI:** 10.1002/ctm2.310

**Published:** 2021-02-01

**Authors:** Le Tao, Xingyu Mu, Haige Chen, Di Jin, Ruiyun Zhang, Yuyang Zhao, Jie Fan, Ming Cao, Zhihua Zhou

**Affiliations:** ^1^ Department of Urology Renji Hospital School of Medicine Shanghai Jiaotong University Shanghai China; ^2^ Department of Urology Shanghai General Hospital School of Medicine Shanghai Jiaotong University Shanghai China; ^3^ Department of Urology Menchao Hepatobiliary Hospital of Fujian Medical University Fuzhou China

**Keywords:** bladder cancer, cell viability, FTO, MAL2, MALAT1, miR‐384, N^6^‐methyladenosine mRNA modification, tumor growth

## Abstract

**Background:**

Nearly a half million people around the world are diagnosed with bladder cancer each year, and an incomplete understanding of its pathogenicity and lack of efficient biomarkers having been discovered lead to poor clinical management of bladder cancer. Fat mass and obesity‐associated protein (FTO) is a critical player in carcinogenesis. We, here, explored the role of FTO and unraveled the mechanism of its function in bladder cancer.

**Methods:**

Identification of the correlation of FTO with bladder cancer was based on both bioinformatics and clinical analysis of tissue samples collected from a cohort of patients at a hospital and microarray data. Gain‐of‐function and loss‐of‐function assays were conducted in vivo and in vitro to assess the effect of FTO on bladder carcinoma tumor growth and its impact on the bladder carcinoma cell viability. Moreover, the interactions of intermediate products were also investigated to elucidate the mechanisms of FTO function.

**Results:**

Bladder tumor tissues had increased FTO expression which correlated with clinical bladder cancer prognosis and outcomes. Both in vivo and in vitro, it played the function of an oncogene in stimulating the cell viability and tumorigenicity of bladder cancer. Furthermore, FTO catalyzed metastasis‐associated lung adenocarcinoma transcript 1 (MALAT1) demethylation, regulated microRNA miR‐384 and mal T cell differentiation protein 2 (MAL2) expression, and modulated the interactions among these processes.

**Conclusions:**

The interplay of these four clinically relevant factors contributes to the oncogenesis of bladder cancer. FTO facilitates the tumorigenesis of bladder cancer through regulating the MALAT/miR‐384/MAL2 axis in m6A RNA modification manner, which ensures the potential of FTO for serving as a diagnostic or prognostic biomarker in bladder cancer.

## Graphical abstract

FTO stimulates tumor growth of bladder cancer through regulating MALAT1 methylation.

MALAT1 and MAL2 interact with miR‐384 for regulating tumor growth of bladder cancer.

FTO promotes bladder cancer tumor growth via MALAT1/miR‐384/MAL2 axis.

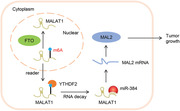

## 1 INTRODUCTION

As one of the most prevalent heterogeneous and malignant cancers in the world, bladder cancer adds approximately 400,000 new diagnoses each year.^1^ Elderly people older than 65 years are typically more vulnerable to developing bladder cancer than individuals younger than 65 years, and males have an approximately four‐fold higher incidence rate of bladder carcinogenesis than do females.^2^ Bladder transitional cell carcinoma is identified in the majority of the patients with bladder cancer (>90%), followed by bladder squamous cell carcinoma (∼5%) and bladder adenocarcinoma (<2%).^3^ In fact, both intrinsic factors (i.e., genetic conditions) and extrinsic factors (i.e., environmental conditions) are involved in the pathogenesis of this multifactorial human cancer type.^4^ Specifically, occupational and environmental exposures to carcinogens like smoking are the most common causative agent of bladder cancer, contributing to 30–50% of the cases.^5^ However, a poor understanding of the pathogenicity of bladder cancer at the molecular level has restricted advances in its clinical management.^6^

Highly accurate and sensitive molecular detection of the onset of bladder cancer is critical for establishing an early diagnosis, estimating a prognosis and predicting recurrence.^7^ Recently, several molecular markers, such as long noncoding RNA (lncRNA), small noncoding RNA (miRNA), and transmembrane proteins, have been reported to participate in pathological alterations and oncogenesis in various human organs.^7^ Metastasis‐associated lung adenocarcinoma transcript 1 (MALAT1), for instance, is linked with a limited prognosis of bladder cancer, facilitating the clinical metastasis and progression of the cancer^8^; miR‐384 is a potential therapeutic target for renal carcinoma due to its function of suppressing cell growth and transformation^9^; mal T cell differentiation protein 2 (MAL2) is recognized as a tumor progression factor in multiple types of human cancers.^10^ Although a large number of these novel biomarkers are promising in their ability to predict the outcomes in cancer, their detailed mechanisms of action and mutual interplay remain not fully understood, and only few of them have been implemented into clinical practice.^11^

Fat mass and obesity‐associated protein (FTO) controls food conversion and energy expenditure, and it is highly connected with body mass index and obesity in humans. Furthermore, it influences adipogenesis.^12^, ^13^ At the same time, FTO regulates mitochondrial biogenesis and oxidative stress through the post‐transcriptional modification of relevant genes.^14^ On one hand, multiple single‐nucleotide polymorphisms in the FTO gene have strong connection with human body weight, and their aberrant regulation can indirectly raise the risk of carcinogenesis.^15^ On the other hand, the substantial role of FTO in cellular metabolism is also considered as an influential factor directly associated with the pathogenesis of cancer.^15^ Several scientists have reported that FTO is overexpressed in various human cancers and that it stimulates cancer cell metabolism, thus inducing tumorigenesis and chemoresistance.^15^ Although it has been demonstrated to play critical roles in various cancers, there is limited knowledge about the specific mechanism of FTO in the tumor initiation of bladder cancer.

Based on the abovementioned previous findings, we designed the present study to analyze the functional roles and molecular mechanism of FTO in the tumorigenesis of bladder cancer. Through successfully establishing FTO‐knockdown and FTO‐overexpressed human bladder cancer cells, we revealed that FTO is oncogenic, as demonstrated by its ability to stimulate cell viability and tumor growth in bladder cells. Furthermore, by exploring the interactions among MALAT1, MAL2, and miR‐384, we also demonstrated that the underlying mechanism of the oncogenic effects of FTO was via regulation of the MALAT1/miR‐384/MAL2 axis. FTO, MALAT1, miR‐384, and MAL2 are all clinically relevant biomarkers in bladder cancer, among which the expression of FTO influences the prognosis of bladder cancer patients. Overall, our findings demonstrate that FTO plays oncogenic roles in the molecular basis of cancer, and they indicate that FTO is a potential diagnostic or prognostic biomarker in bladder cancer.

## 2 METHODS

### 2.1 Bioinformatic analyses

The gene expression data of bladder tumor tissue (n = 41) and normal bladder tissue (n = 9) were downloaded from the GSE3167 database. Kaplan–Meier analysis was downloaded from TCGA dataset to quantify the disease‐free survival and overall survival of bladder cancer patient. N^6^‐methyladenosine (m^6^A) sequencing (m6A‐seq) and RNA sequencing (RNA‐seq) were performed as previously described,^16^ and these data were uploaded to Gene Expression Omnibus (http://www.ncbi.nlm.nih.gov/geo/) under accessible number of GSE150239.

### 2.2 Patients and tissue sample collection

Paired (n = 25 each) tumor and corresponding adjacent noncancerous fresh tissues obtained from bladder cancer patients admitted to Renji Hospital during October 2012 to March 2017 were designated as cohort 1. Microarrays of the human bladder cancer tissues obtained from Outdo Biotech Co., Ltd. (Shanghai, China), were designated as cohort 2. Table 1 shows the clinical parameters of the included patients. None of the included patients were exposed to radiotherapy or chemotherapy prior to undergoing surgery. All ethical regulations set by the ethics committee of Renji Hospital were followed during this study. All patients were duly informed before the study, and a written consent was obtained.

**TABLE 1:** Clinicopathological features of bladder cancer patients in two cohorts

Clinicopathological parameter	Cohort 1 (number of patients)	Cohort 2 (number of patients)
**Age**		
≤55	11	67
>55	14	77
**Sex**		
Female	10	55
Male	15	89
**Tumor size (cm)**		
≤4	12	72
>4	13	72
**Smoke**		
Yes	14	72
No	11	72
**Pathologic stages**		
pTa‐T1	11	62
pT2‐T4	14	82

p < 0.05 represents statistical significance (chi‐square test).

### 2.3 Immunohistochemistry staining

FTO and MAL2 protein analysis was performed by immunohistochemistry (IHC) staining of bladder cancer tissue microarrays following standard protocol using antibodies (1:100 dilution) against FTO (ab124892, Abcam, Cambridge, MA, USA) and MAL2 (bs‐7175R, BIOSS, Woburn, MA, USA). The H‐score system, based on the percentage of positively stained cells, was used to assess immunoreactivity, and this assessment was performed by two investigators. Based on the immunoreactivity scores, the bladder cancer patients were categorized into the low expression (H‐score < 50%) group and the high expression (H‐score > 50%) group.

### 2.4 Cell culture

The human bladder cancer 5637, J82, 253J, T24, and SCABER cell lines, as well as a non‐cancerous human bladder cell line (SV‐HUC‐1), were obtained from the Shanghai Institute of Biochemistry and Cell Biology (Shanghai, China) and cultured at 95% humidity, 5% CO_2_, and 37˚C in DMEM or RPMI‐1640 medium (Hyclone) with 10% fetal bovine serum (Gibco) and 1.0% penicillin‐streptomycin (Solarbio) solutions.

### 2.5 Plasmid construction and cell transfection

The hsa‐miR‐384 mimics (5′‐AUUCCUAGAAAUUGUUCAUA‐3′), hsa‐miR‐384 inhibitor (5′‐UAUGAACAAUUUCUAGGAAU‐3′), and negative control (NC, 5′‐UUCUCCGAACGUGUCACGUTT‐3′) were synthesized by Ribo Bio Corporation (Guangzhou, China) and transfected using Lipofectamine 2000 reagent (Invitrogen).

The short hairpin RNAs (shRNAs) against human FTO, MALAT1, MAL2, YTHDF2, YTHDF3, or YTHDC2 (Table 2 for sequences) and scramble shRNA acting as negative control were cloned into the pLKO.1 vector (Addgene). The cDNA encoding FTO was inserted into pLVX‐Puro plasmids (Clontech) to obtain the vector expressing pLVX‐Puro‐FTO, and the empty vector acted as negative control. The 293T cells (ATCC, Manassas, VA, USA) were transfected with the constructs by using Lipofectamine 2000 reagent (Invitrogen) as well. Subsequently, the cells were incubated in a CO_2_ incubator at 37°C for 48 hours, which was followed by the collection of recombined vectors being performed. 253J, T24, and 5637 cells were infected with the recombined vectors as previously described,^17^ and the expression of FTO was measured by quantitative real‐time PCR (Q‐PCR) and western blot at 72 hours after infection.

**TABLE 2:** Interfering RNA sequences used in this study

Gene	Sequences (5′‐3′)
Human FTO shRNA‐1	GGAGCTCCATAAAGAGGTT
Human FTO shRNA‐2	CCTGAACACCAGGCTCTTT
Human FTO shRNA‐3	GCAGCATACAACGTAACTT
Human MALAT1 shRNA‐1	GCCCGAGACTTCTGTAAAG
Human MALAT1 shRNA‐2	GAGTTGTGCTGCTATCTTA
Human MALAT1 shRNA‐3	GCTCTAAATTGTTGTGGTT
Human MAL2 shRNA‐1	GCATGTTCCTCTCTGGCAT
Human MAL2 shRNA‐2	GCATTGCAATACAACCATA
Human MAL2 shRNA‐3	GCTTGTTATGGTTGCAGTT
Human YTHDF2 shRNA‐1	GCACAGAAGTTGCAAGCAA
Human YTHDF2 shRNA‐2	GCACAGAGCATGGTAACAA
Human YTHDF3 shRNA‐1	CCTATGGACAAATGAGTAA
Human YTHDF3 shRNA‐2	GCAGTGGTATGACTAGCAT
Human YTHDC2 shRNA‐1	GCGACTCAACAATGGCATA
Human YTHDC2 shRNA‐2	GGAAATGGATGCTTGCCTT

### 2.6 Cell proliferation measurement

Cell proliferation was detected by the cell counting kit‐8 (CCK8) assay (SAB Biotech, USA). Approximately 3 × 10^3^ cells were cultured in each well of a 96‐well plate for 12 hours. At 0, 24, 48, and 72 hours, CCK8 was mixed with the cells in each individual well, and the cells were subsequently cultured for 1 hour. Then, the absorbance of each well was measured at 450 nm.

### 2.7 RNA extraction, cDNA synthesis, and Q‐PCR

Trizol reagent (Invitrogen) was used to extract the total RNA from the bladder cancer tissues or cell lines. Transcriptor cDNA synthesis kit (Roche Diagnostics Corporation, Indianapolis, IN, USA) was used for the reverse transcription of the cDNA. We used SYBR Green Q‐PCR with specific primers to compare FTO, MALAT1, MAL2, miR‐384, and pri‐miR‐384 expression (Table 3 for sequences). The relative expression of target gene was quantified by the comparative 2^–ΔΔCt^ method, with GAPDH or U6 serving as the internal control.

**TABLE 3:** Primes sequences for Q‐PCR used in this study

Gene	Sequences (5′‐3′)
FTO‐forward	ACCTCCAGCATTAGATTC
FTO‐reverse	GAAACTACCGCATTTACC
MALAT1‐forward	TTTCTTCCTGCTCCGGTTC
MALAT1‐reverse	TTTCAGCTTCCAGGCTCTC
MAL2‐forward	ACTTACCTTTGGAGGAATG
MAL2‐reverse	TCTTGCTCAGTTGTTAGAC
GAPDH‐forward	AATCCCATCACCATCTTC
GAPDH‐reverse	AGGCTGTTGTCATACTTC
miR‐384‐forward	CGCGCGATTCCTAGAAATTG
miR‐384‐reverse	AGTGCAGGGTCCGAGGTATT
pri‐miR‐384‐forward	TTCAGGGCTTTGAACACGC
pri‐miR‐384‐reverse	GGATGGGGCTTTAGAGGATC
U6‐forward	CTCGCTTCGGCAGCACA
U6‐reverse	AACGCTTCACGAATTTGCGT

### 2.8 Protein extraction and western blot analysis

Radioimmunoprecipitation lysis buffer was used for extracting total protein. Protein separation was done by running extracted proteins on sodium dodecyl sulfate‐polyacrylamide gel electrophoresis and then transferred onto nitrocellulose membranes (Millipore, Bedford, USA), followed by 5% skim milk blocking with antibodies against FTO (1:1000, ab124892; Abcam), MAL2 (1:500, ab75347; Abcam), and GAPDH (1: 2000, #5174; Cell Signaling Technology, USA).

### 2.9 Analysis of m^6^A content

Trizol reagent (Invitrogen) was used to extract the total RNA. Poly(A)^+^ RNA was purified using GenElute mRNA Miniprep Kit (Sigma, Louis, MO). The content of m6A in total RNA was estimated by using m^6^A RNA Methylation Assay Kit (ab185912; Abcam). Briefly, 80 μL of binding solution and 200 ng of sample RNA were added into each designated well and then incubated at 37°C for 90 minutes for RNA binding. Wash each well three times with wash buffer. 50 μL of the diluted capture antibody was added into each well and then incubated at room temperature for 60 minutes. Each well was incubated with detection antibody and enhancer solution at room temperature for 30 minutes subsequently. Finally, the wells were incubated with developer solution in the dark for 1 to 10 minutes at 25°C. Reaction was stopped with stop solution and determined using a microplate reader at 450 nm wavelength within 2–10 minutes.

### 2.10 Luciferase reporter assay

The wide‐type and mutant 5′‐End sequences of MALAT1 were synthesized at Generay Technologies (Shanghai, China) and inserted into the upstream of pGL3‐basic firefly luciferase vector (Promega). Then, the pGL3‐MALAT1‐5′‐End and pRL‐TK renilla (Promega) luciferase reporter vector were cotransfected into the bladder cancer cell lines, which were transduced with FTO silencing or FTO‐expressing vector. MALAT1 sequences harboring a putative miR‐384 binding site and 3′‐UTR sequences of MAL2 as well as their mutant sequences were inserted into the pGL3 vector. Then, the wild‐type or mutant pGL3‐MALAT1 or pGL3‐MAL2 and pRL‐TK renilla (Promega) luciferase reporter vector were cotransfected into the bladder cancer cells, which were transfected with miR‐384 mimics or NC. Luciferase activity was assessed using a Dual‐Luciferase Reporter Assay system (Promega) at 48 hours post‐transduction.

### 2.11 Measurement of mRNA stability

The bladder cancer cells were treated with transcriptional inhibitor actinomycin D (APExBIO Technology LLC, Houston, TX, USA, 0.2 mM) for 30 minutes and harvested. At 0, 3, and 6 hours, the samples were collected for total RNA extraction and cDNA synthesis, which were performed according to the methods described above. Q‐PCR was performed for quantification of mRNA levels.

### 2.12 RNA immunoprecipitation assay

Magna RNA immunoprecipitation (RIP) RNA‐Binding Protein Immunoprecipitation kit (Millipore) was used for the RIP assay following the manufacturer's instructions. Cells were prepared using RIP lysis buffer, and the RNA‐protein complexes were conjugated with anti‐YTHDF2 (Abcam, ab220163, 1:30), anti‐YTHDF3 (Abcam, ab220161, 1:30), anti‐YTHDC2 (Abcam, ab220160, 1:30) or anti‐IgG antibody (Abcam, ab172730, 1:30) overnight at 4°C and washed with RIP‐wash buffer for 10 minutes at 4°C and then RIP‐lysis buffer for 5 minutes at 4°C. The co‐precipitated RNAs were purified using phenol:chloroform:isoamyl alcohol and subjected to Q‐PCR.

### 2.13 Xenograft study in a mice model

Four‐ to 5‐week‐old male nude mice (n = 5 per group, n = 20 mice total) were purchased from Shanghai Laboratory Animal Company (Shanghai, China). Approximately 5 × 10^6^ 253J and 5637 cells infected with indicated vectors were injected subcutaneously into the flank of the mice. To calculate the tumor growth, the tumor volumes of the mice were measured every 3 days. On day 33 after cell implantation, the mice were sacrificed, and the tumor xenografts were collected. Then, the tumor xenograft was weighed. All experimental procedures were approved by the Institutional Animal Care and Use Committee of Renji Hospital.

### 2.14 Immunofluorescence microscopy

After being fixed and permeabilized, the tissues collected from the xenograft tumor underwent 1% bovine serum albumin blocking in phosphate buffer saline (PBS) for 30 minutes and incubation with anti‐Ki67 (Abcam; ab245113, 1:1000) and Alexa Fluor 488‐labeled Goat Anti‐Mouse IgG (H+L) (Beyotime Biotechnology; A0428, 1:500) antibodies. DAPI (Beyotime Biotechnology; C1002, 1:500) labeling was used to visualize cell nuclei. Then, visualization of the positively stained cells was performed using confocal laser scanning microscope (Leica Microsystems, Inc., USA).

### 2.15 Data analyses

All of the experiments and assays were conducted in triplicate, and data were shown as mean ± standard deviation. Statistical analysis was performed using GraphPad Prism 7.0 statistical software (GraphPad Software, San Diego, CA, USA). The Cox proportional‐hazards regression model and Kaplan–Meier method were used to assess overall survival, and the log‐rank test was performed to determine differences between groups. We used the Mann–Whitney U test or unpaired *t*‐test to perform comparisons between groups, and one‐way and two‐way ANOVA with Bonferroni's post hoc test were used to perform multiple comparisons. Statistically significant differences were determined based on p < 0.05.

## 3 RESULTS

### 3.1 FTO is an oncogenic gene and is correlated with bladder cancer prognosis

**FIGURE 1:**
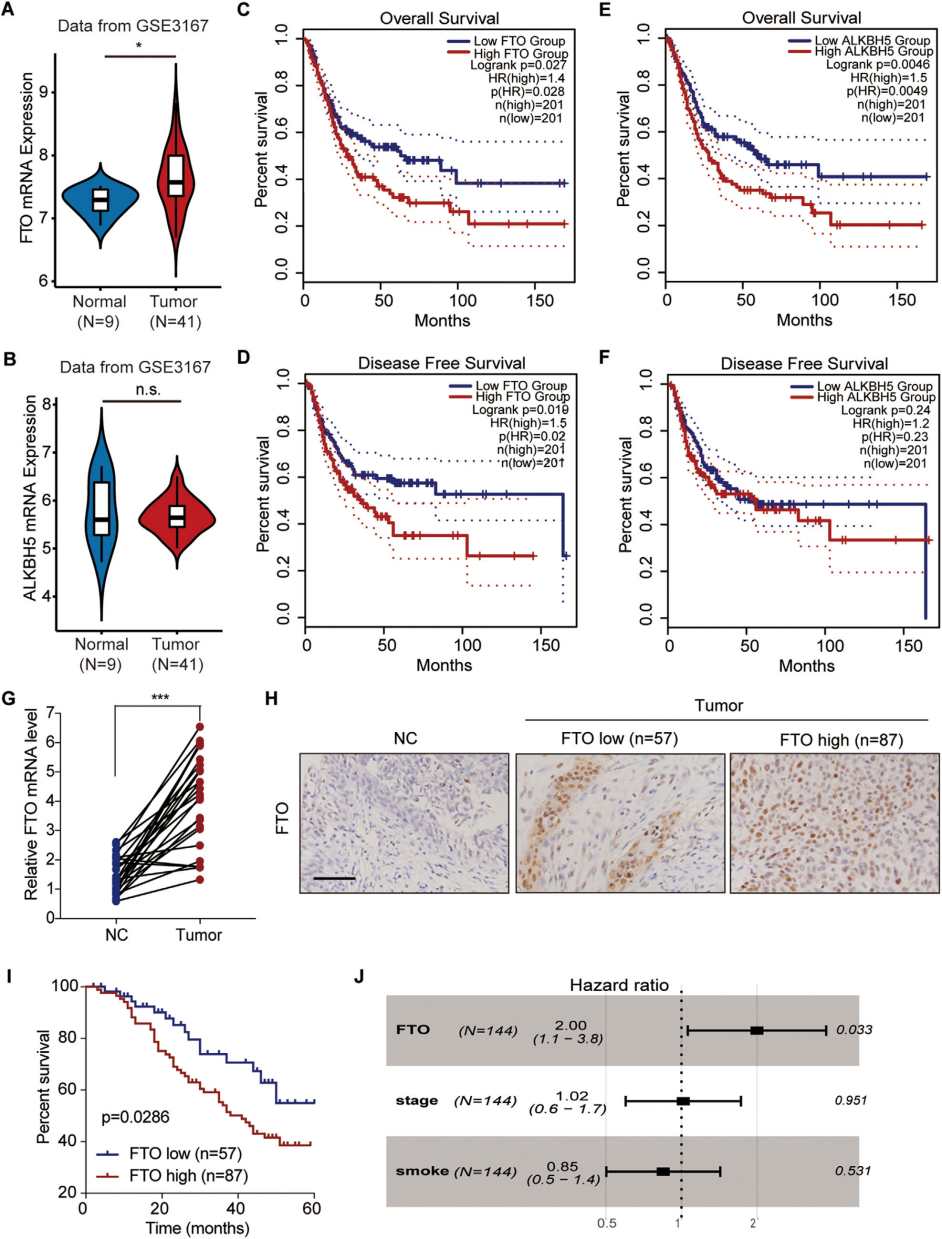
Correlation between FTO and bladder cancer prognosis. The expression levels of ALKBH5 (A) and FTO (B) in the GSE3167 bladder cancer database, with a comparison between tumor (T; n = 41) and normal (N; n = 9) tissues. The overall survival rate (C, E) and disease‐free survival rate (D, F) of bladder cancer patients (n = 402) in the TCGA database, with a comparison between the low and high ALKBH5 or FTO expression groups. (G) The relative mRNA expression levels of FTO in the bladder cancer patient tissues from cohort 1, with a comparison between the normal (NC) and tumor (n = 25 for each) paired bladder tissues. (H) The protein expression of FTO based on IHC scores of the bladder cancer tissue microarrays from cohort 2, with a comparison among the normal (NC; n = 10), low expression (n = 57), and high expression (n = 87) bladder tissue groups. Scale bar: 100 μm. (I) Kaplan–Meier plot for the overall survival of bladder cancer patients in cohort 2 based on the FTO protein levels according to the IHC score. (J) Multivariate regression analysis for hazard ratios of FTO protein expression, tumor stage, and smoking status in the bladder cancer patients in cohort 2 (n = 144). (A, B, G) The Mann–Whitney U test was used for comparisons between two groups. (C–F, I) The log‐rank test was used for comparisons between three or more groups. ***P < 0.001

In the bioinformatic analysis of the GEO database, we detected that both the ALKBH5 and FTO genes were connected with bladder cancer. Specifically, only the FTO gene, and not the ALKBH5 gene, was highly overexpressed in the tissue of bladder cancer (Figures 1A and 1B). Furthermore, the FTO expression levels were markedly correlated with both the overall (Figure 1C) and disease‐free (Figure 1D) survival rates in the bladder cancer patients from the TCGA database, which splits the patients into separate groups based on the median value, whereas ALKBH5 expression showed significant correlation with the overall survival rate of the patients only (Figures 1E and 1F). Therefore, we focused on FTO for further investigation.

From the analysis of the tissues collected from cohort 1, we identified that the mRNA expression of FTO increased by 2.8‐fold in the tumor tissues of patients with bladder cancer compared to their normal tissues (p < 0.001; Figure 1G). We further detected the FTO protein staining in bladder cancer patients of cohort 2 as described in Methods (Figure 1H). IHC staining indicated that in comparison with normal tissues, FTO protein expression was increased in bladder cancer tissue (Figure 1H). In addition, the overall survival rate was decreased in the bladder cancer patients with a high FTO expression compared to those with a low FTO expression (Figure 1I). Analysis of the clinical characteristics of the patients in cohort 2 indicated that FTO protein expression was closely associated with various clinicopathological features of bladder cancer. FTO expression and smoking status (p = 0.0267) and pathologic stage (p = 0.0262) of bladder cancer patients were significantly correlated with each other (Table 4). Based on the multivariate analysis, FTO protein expression was identified as an independent predictor (p = 0.033) of bladder cancer (Figure 1J).

**TABLE 4:** Clinicopathological features of bladder cancer patients and FTO expression

	FTO		
Clinicopathological parameter	Low group, number of patients	High group, number of patients	*p* value
**Age**			0.0593
≤55	21	46	
>55	36	41	
**Sex**			0.6664
Female	23	32	
Male	34	55	
**Tumor size (cm)**			0.1251
≤4	33	39	
>4	24	48	
**Smoke**			0.0267
Yes	22	50	
No	35	37	
**Pathologic stages**			0.0262
pTa‐T1	31	31	
pT2‐T4	26	56	

p < 0.05 represents statistical significance (chi‐square test).

### 3.2 FTO stimulates cell viability and tumor growth of bladder cancer through regulating MALAT1 methylation

**FIGURE 2:**
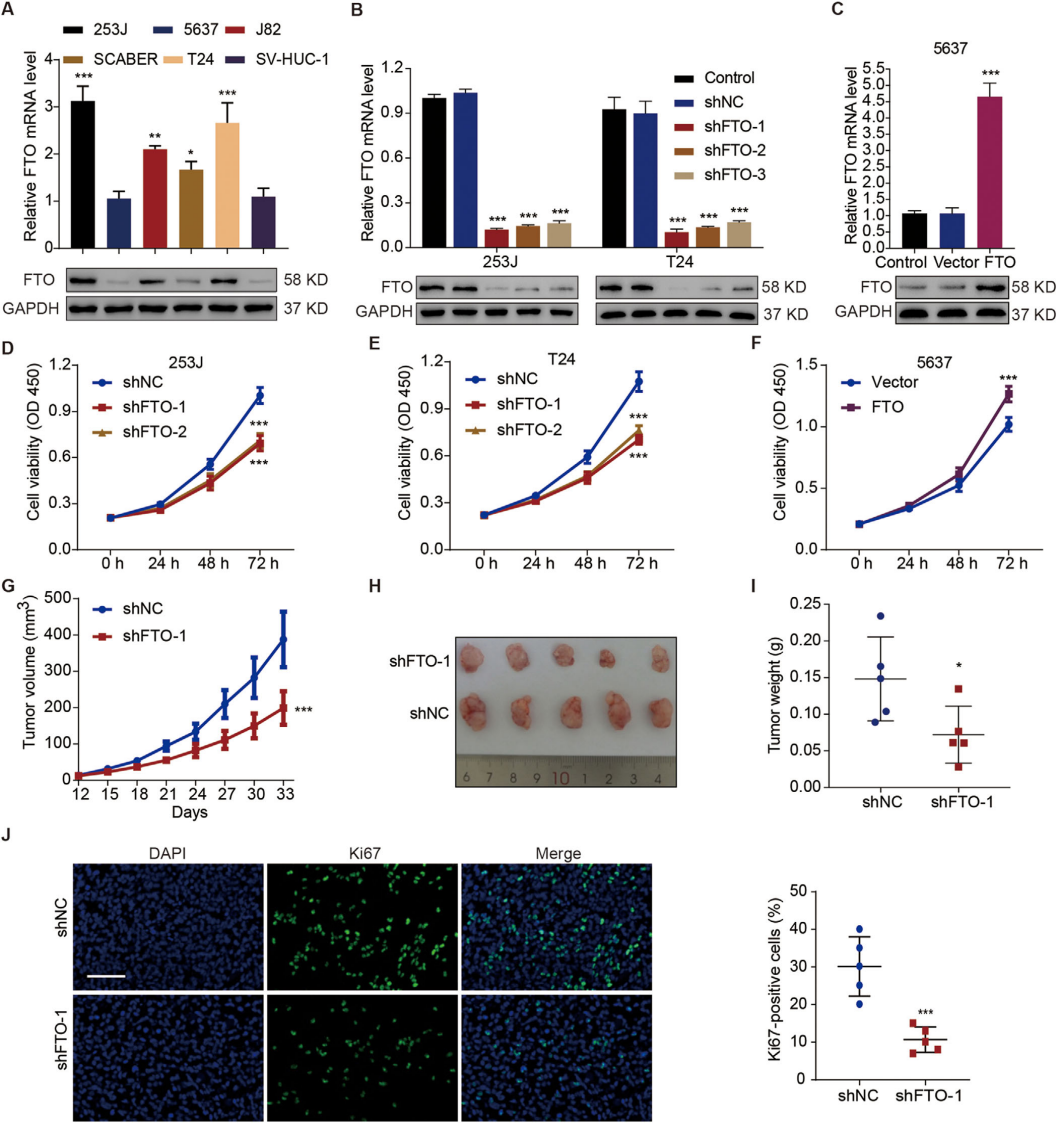
Effects of FTO on cell viability and tumor growth in bladder cancer. (A) The relative mRNA and protein levels of FTO in various bladder cancer cell lines (253J, 5647, J82, SCABER, and T24) and in a normal bladder cell line (SV‐HUC‐1). The relative mRNA and protein levels of FTO in 253J (B), T24 (B), and 5637 cells (C) at 72 hours after being transduced with the indicated constructs. Viability of 253J (D), T24 (E), and 5637 cells (F) transduced with the indicated constructs. (G) Tumor volumes of the xenografts of mice (n = 5 per group) injected with 253J cells transduced with the indicated constructs. Representative tumor photographs (H) and tumor weights (I) at day 33. (J) Immunofluorescence staining for Ki‐67 in the xenograft tumors. Scale bar: 100 μm. One‐way ANOVA (A, C) and two‐way ANOVA (B, D–G) followed by Bonferroni's post hoc test were used to perform comparisons among three or more groups. (I and J) An unpaired *t*‐test was used to perform comparisons between two groups. *p < 0.05, ***p < 0.001 compared with SV‐HUC‐1, shNC, or Vector

The mRNA and protein expression amounts of FTO were quantified in human normal bladder cell and bladder cancer cell lines derived from humans. The 253J and T24 cells exhibited the highest expression levels of FTO, whereas the 5637 cells showed the lowest expression levels of FTO (Figure 2A). Therefore, knockdown of FTO was established in 253J and T24 cells (Figure 2B), and overexpression of FTO was accomplished in 5637 cells (Figure 2C).

Our study subsequently determined the functions of FTO in bladder cancer both in mammalian cells and xenograft of tumor tissues obtained from mice. At 72 hours, FTO‐knockdown significantly (p < 0.001) inhibited cell viability, by 30.1% and 31.8%, in 253J (Figure 2D) and T24 (Figure 2E) cells, respectively, whereas FTO overexpression significantly (p < 0.001) stimulated cell viability, by 1.2‐fold, in 5647 cells (Figure 2F). Furthermore, through the establishment of a xenograft mice model by injecting FTO‐knockdown 253J cells, we observed on day 33 after cell implantation that tumor growth (p < 0.001; Figure 2G) and tumor weight were significantly decreased, by 48.7% and 51.2%, respectively (p < 0.05; Figures 2H and 2I). Meanwhile, knockdown of FTO decreased the expression of Ki67 by 64.6% in tumor tissues (Figure 2J). These findings suggest that FTO may act as an oncogene in the carcinogenesis of bladder cancer.

**FIGURE 3:**
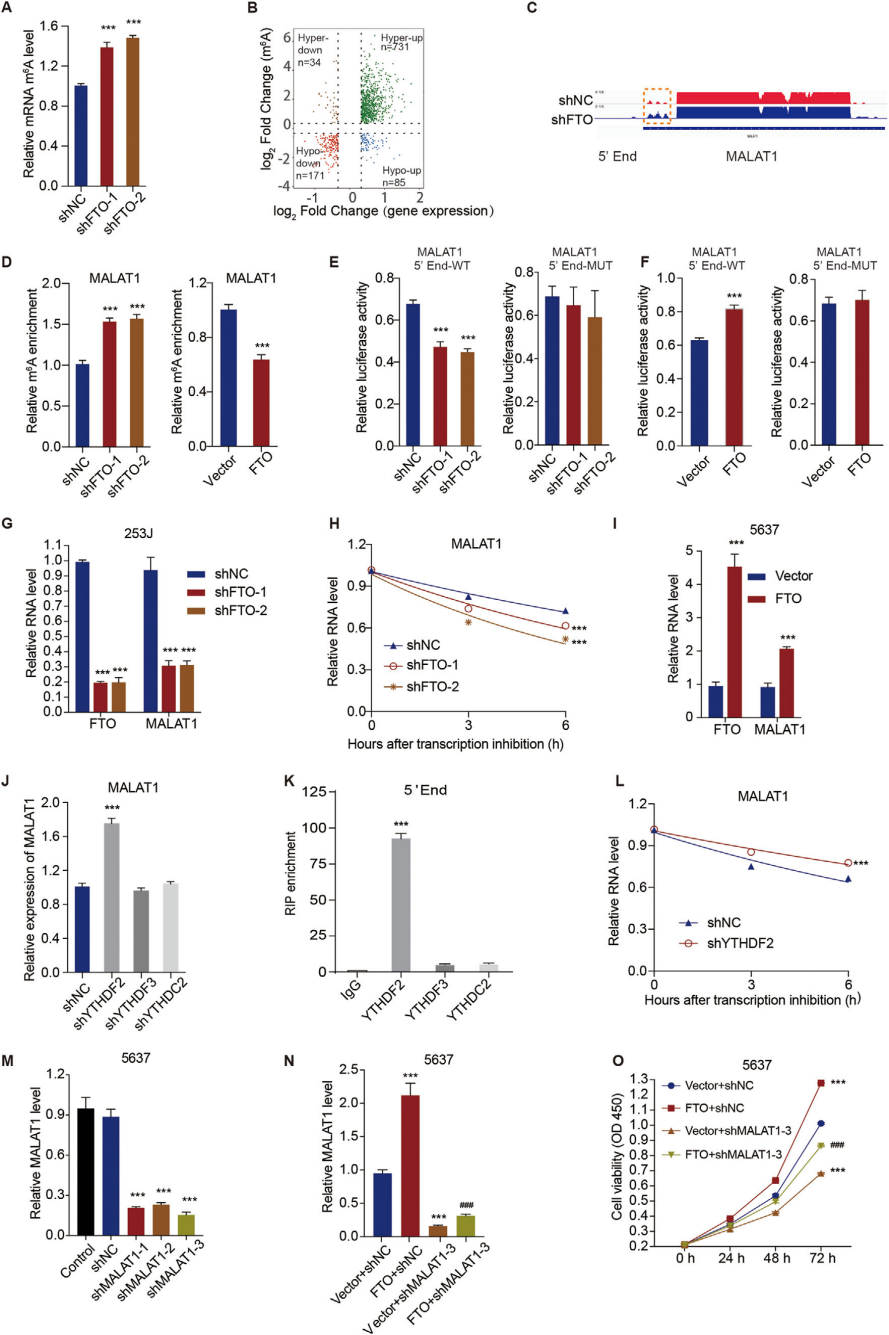
FTO promotes cell viability of bladder cancer cells through regulation of MALAT1 mRNA m^6^A modification and stability. (A) The mRNA levels of global m^6^A in control and FTO‐knockdown 253J cells. (B) Volcano plots of the distinct m^6^A peaks (fold change ≥ 2 and p *< *0.05). (C) The relative abundance of m^6^A sites along the MALAT1 mRNA in control and FTO‐knockdown 253J cells. The blue rectangles represent a decrease in the abundance of m^6^A peaks. (D) The relative m^6^A levels of MALAT1 5′‐end in FTO‐knockdown 253J cells (left panel) and FTO overexpression 5637 cells (right panel). (E and F) MALAT1 5′‐end enrichment in WT and mutated (MUT) 253J cells (E) and 5637 cells (F) transduced with the indicated constructs for FTO. (G) The relative mRNA levels of MALAT1 in FTO‐knockdown 253J cells. (H) The half‐life curve of MALAT1 transcription in FTO‐knockdown 253J cells. (I) The relative mRNA levels of MALAT1 in FTO overexpression 5637 cells. (J) The mRNA levels of MALAT1 in 253J cells transduced with the indicated constructs. (K) RNA immunoprecipitation of YTHDF2 and MALAT1 mRNA, with IgG as the control. (L) The half‐life curve of MALAT1 transcription in YTHDF2‐knockdown 253J cells. (M and N) The expression level of MALAT1 in 5637 cells transduced with the indicated constructs. (O) Viability of 5637 cells transduced with the indicated constructs. One‐way ANOVA (A, D‐left panel, E, J, K, M, N) and two‐way ANOVA (G‐I, L, O) followed by Bonferroni's post hoc test were used to perform comparisons of three or more groups. (D‐right panel, F) An unpaired *t*‐test was used to perform comparisons between two groups. ***p < 0.001 compared with shNC, Vector, IgG, or Vector+shNC. ^###^p < 0.001 compared with FTO+shNC

To recognize the potential mRNA targets of FTO in bladder cancer cells, we next performed transcriptome‐wide sequencing of FTO regulated m^6^A (m^6^A‐seq) and RNA‐seq assays on FTO‐knockdown and control 253J cells. Notably, the m^6^A level increased by 1.5‐fold in the polyadenylated RNA (poly(A) RNA) of the FTO‐knockdown 253J cells compared to that of the 253J cells without FTO knockdown (Figure 3A). The m^6^A‐seq data demonstrated that on a transcription level m^6^A was hypermethylated globally after the FTO knockdown in 253J cells. A total of 2687 and 1777 peaks demonstrated a marked increase and a marked decrease, respectively, in m^6^A modifications (Table S1) in the FTO‐knockdown cells in comparison with the control cells, and they were therefore designated as hypermethylated and hypomethylated m^6^A peaks. Altogether 2657 and 2332 genes exhibited a marked increase and a marked decrease, respectively, in mRNA expression in the FTO‐knockdown cells in comparison with the control cells (Table S2). In the RNA‐seq analysis of the FTO‐knockdown cells, we identified 34 hypermethylated m^6^A genes with downregulated mRNA transcripts (p < 0.05, Hyper‐down) and 731 hypermethylated m^6^A genes with upregulated mRNA transcripts (p < 0.05, Hyper‐up) (Figure 3B). Considering the role of FTO as an m^6^A silencer, mRNA transcripts with hypermethylated peaks of m^6^A in 253J FTO‐knockdown cells were the potential targets. In the m^6^A‐seq data analysis, we noticed that the m^6^A RNA modification level of MALAT1 is most significantly increased in bladder cancer cells after knockdown of FTO, and it has been previously reported that MALAT1 may mediate the progression of bladder cancer (Figure 3C).^17^, ^18^ Therefore, we analyzed MALAT1 for further validation.

Moreover, we found that FTO silencing in bladder cancer cells could significantly (p < 0.001) upregulate, by 1.5‐fold, the relative m^6^A level of MALAT1 5′‐End (Figure 3D), whereas the overexpression of FTO could significantly (p < 0.001) decrease, by 24.3%, the relative m^6^A level of MALAT1 5′‐End (Figure 3D). Meanwhile, we constructed WT and mutation MALAT1 5′‐end luciferase reporter plasmids. In the mutation MALAT1 5′‐end luciferase reporter plasmids, m^6^A was replaced by T. As expected, knockdown of FTO significantly decreased, by 33.8%, luciferase activity in the WT MALAT1 reporter plasmid (Figure 3E), and overexpression of FTO significantly increased, by 1.33‐fold, luciferase activity in the WT MALAT1 reporter plasmid (Figure 3F). However, knockdown and overexpression of FTO did not have significant effects on luciferase activity in the mutated MALAT1 reporter plasmid (Figures 3E, 3F). Overall, in bladder cancer cells, knockdown of FTO significantly (p < 0.001) decreased the relative expression level (Figure 3G) and half‐life (Figure 3H) of MALAT1 mRNA, whereas FTO overexpression significantly increased, by 2.2‐fold, the relative expression level and half‐life of MALAT1 mRNA (Figure 3I).

Furthermore, the genes encoding common m^6^A reader proteins, including YTHDF2, YTHDF3, and YTHDC2, were silenced in the 253J cells (Figure S1), and we found that YTHDF2 knockdown significantly (p < 0.001) increased, by 1.8‐fold, the transcriptional expression level of MALAT1 (Figure 3J). Results of the RIP assay indicated that YTHDF2 can bind to MALAT1 5′‐end but not to YTHDF3 or YTHDC2 (Figure 3K). The RNA stability assay showed that YTHDF2‐knockdown significantly increased, by 1.2‐fold, the half‐life of MALAT1 mRNA (Figure 3L). Additionally, through establishing MALAT1‐knockdown in 5637 cells (Figures 3M and 3N), we revealed that MALAT1 knockdown could substantially (p < 0.001) block the FTO‐induced increase in cell viability in 5637 cells (Figure 3O).

### 3.3 MALAT1 and MAL2 interact with miR‐384 to regulate cell viability in bladder cancer

**FIGURE 4:**
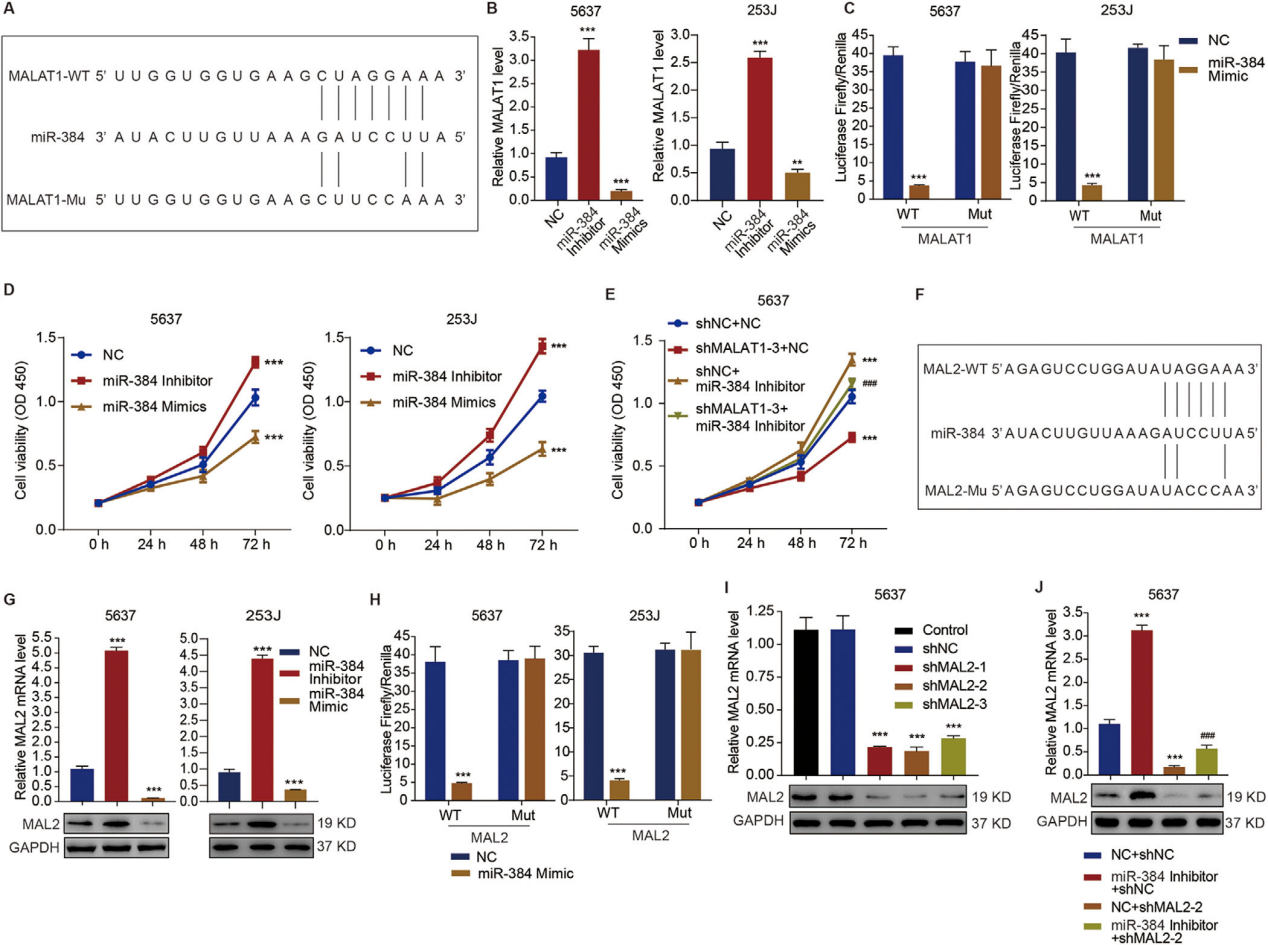
Roles of MALAT1 and MAL2 in miR‐384‐mediated cell viability of bladder cancer cells. (A) The predictive binding between miR‐384 and MALAT1. (B) The expression levels of MALAT1 in 5637 and 253J cells with miR‐384 inhibitor or mimics. (C) Luciferase activity of WT and MUT MALAT1 reporter plasmids in 5637 and 253J cells with miR‐384 mimics. (D and E) Cell viability of 5637 or 253J cells transduced with the indicated constructs. (F) The predictive binding between miR‐384 and MAL2. (G) The mRNA and protein expression levels of MAL2 in 5637 and 253J cells with miR‐384 inhibitor or mimics. (H) Luciferase activity of WT and MUT MAL2 reporter plasmids in 5637 and 253J cells with miR‐384 mimics. (I and J) The expression level of MAL2 in 5637 cells transduced with the indicated constructs. One‐way ANOVA (B, G, I, J) and two‐way ANOVA (C, D, E, H) followed by Bonferroni's post hoc test were used to perform the comparisons. **p < 0.01, ***p < 0.001 compared with NC, shNC, or NC+shNC. ^###^p < 0.001 compared with shNC+miR‐384 inhibitor

Through bioinformatic analysis and sequence alignment, the interaction binding sites between miR‐384 and MALAT1 were predicted from starBase v2.0 (http://rna.sysu.edu.cn/encori/index.php) (Figure 4A). We further established the miR‐384 inhibition or mimic in bladder cancer cells, and we found that miR‐384 inhibitor substantially (p < 0.001) elevated the transcriptional expression level of MALAT1, by 3.7‐fold and 2.8‐fold, in 5637 and 253J cells, respectively, whereas miR‐384 mimics had the opposite effect (Figure 4B). Moreover, miR‐384 mimics substantially (p < 0.001) reduced the luciferase activity of wide‐type MALAT1 reporter plasmid by 90.3% and 89.4% in 5637 and 253J cells, respectively, while miR‐384 mimics had no effect on the luciferase activity of mutant MALAT1 reporter plasmid (Figure 4C). Meanwhile, the viability of 5637 and 253J cells at 72 hours was substantially (p < 0.001), by 1.3‐fold and 1.4‐fold, respectively, stimulated by miR‐384 inhibitor and suppressed by miR‐384 mimics, by 29.8% and 39.4‐fold, respectively (Figure 4D). Furthermore, we also observed that MALAT1 knockdown substantially (p < 0.001) counteracted the promotive effect of miR‐384 inhibitor on cell viability (Figure 4E). Similarly, the interaction binding sites between miR‐384 and MAL2 were predicted from PITA microRNA database (https://genie.weizmann.ac.il/pubs/mir07/mir07_dyn_data.html) (Figure 4F). In the 5637 and 253J cells, miR‐384 inhibitor substantially (p < 0.001) upregulated both the mRNA and protein expression of MAL2, whereas the miR‐384 mimics substantially (p < 0.001) downregulated the mRNA and protein expression levels of MAL2 (Figure 4G). Moreover, miR‐384 mimics also substantially (p < 0.001) reduced the enrichment of MAL2 3′‐UTR in 5637 and 253J cells, by 87.4% and 86.6%, respectively, whereas mutation of MAL2 3′‐UTR counteracted its effect (Figure 4H). Meanwhile, based on the establishment of MAL2 knockdown in 5637 cells (Figure 4I), we detected that MAL2 knockdown substantially (p < 0.001) counteracted the stimulatory effects of miR‐384 inhibitor on MAL2 expression (Figure 4J). The data suggest that MALAT1 may act as a RNA sponge to interact with miR‐384 and further modulation MAL2 expression and promote bladder cancer proliferation.

### 3.4 FTO promotes bladder cancer cell viability and tumor growth via the MALAT1/miR‐384/MAL2 axis

**FIGURE 5:**
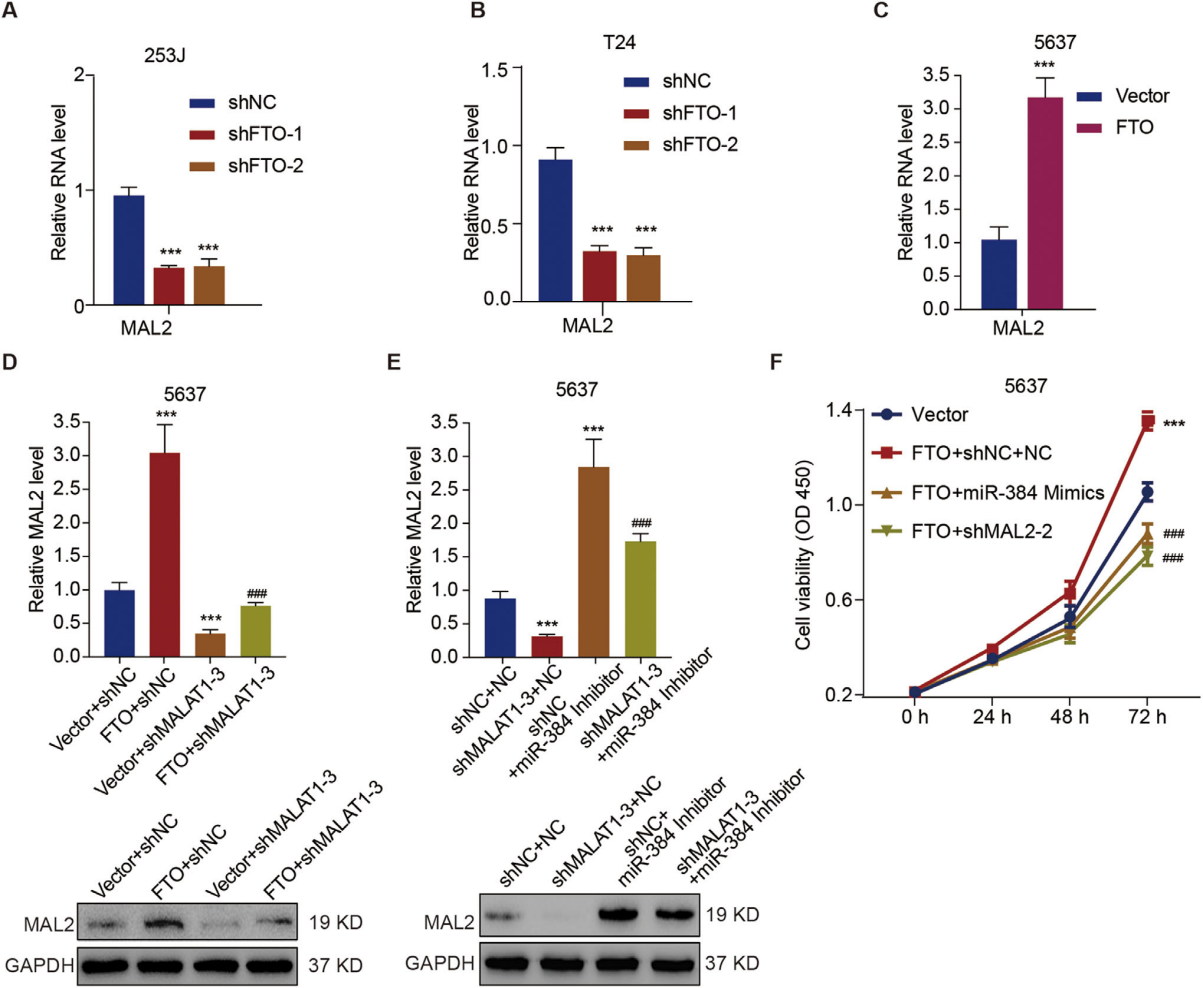
Effects of the MALAT1/miR‐384/MAL2 axis on the promotion of bladder cancer cell viability by FTO. The relative mRNA levels of MAL2 in FTO‐knockdown 253J cells (A) and FTO‐knockdown T24 cells (B). (C) The relative mRNA levels of MAL2 in FTO overexpression 5637 cells. (D and E) The expression level of MAL2 in 5637 cells transduced with the indicated constructs. (F) Cell viability of 5637 cells transduced with the indicated constructs. Unpaired *t*‐test (C) and one‐way ANOVA (A, B, D, E) and two‐way ANOVA (F) followed by Bonferroni's post hoc test were used to perform the comparisons. ***p < 0.001 compared with shNC, Vector, Vector+shNC, or shNC+NC. ^###^p < 0.001 compared with FTO+shNC, shNC+miR‐384 inhibitor, or FTO+shNC+NC

We further investigated the functional roles of FTO in the MALAT1/miR‐384/MAL2 axis. The knockdown of FTO in 253J cells (Figure 5A) and T24 cells (Figure 5B) significantly (p < 0.001) downregulated MAL2 at the transcriptional level. In contrast, FTO overexpression in 5637 cells significantly upregulated the mRNA level of MAL2 by 3.0‐fold (Figure 5C). Meanwhile, MALAT1 knockdown significantly (p < 0.001) counteracted the upregulation of the mRNA and protein levels of MAL2 induced by FTO overexpression (Figure 5D) and miR‐384 inhibitor (Figure 5E).

**FIGURE 6:**
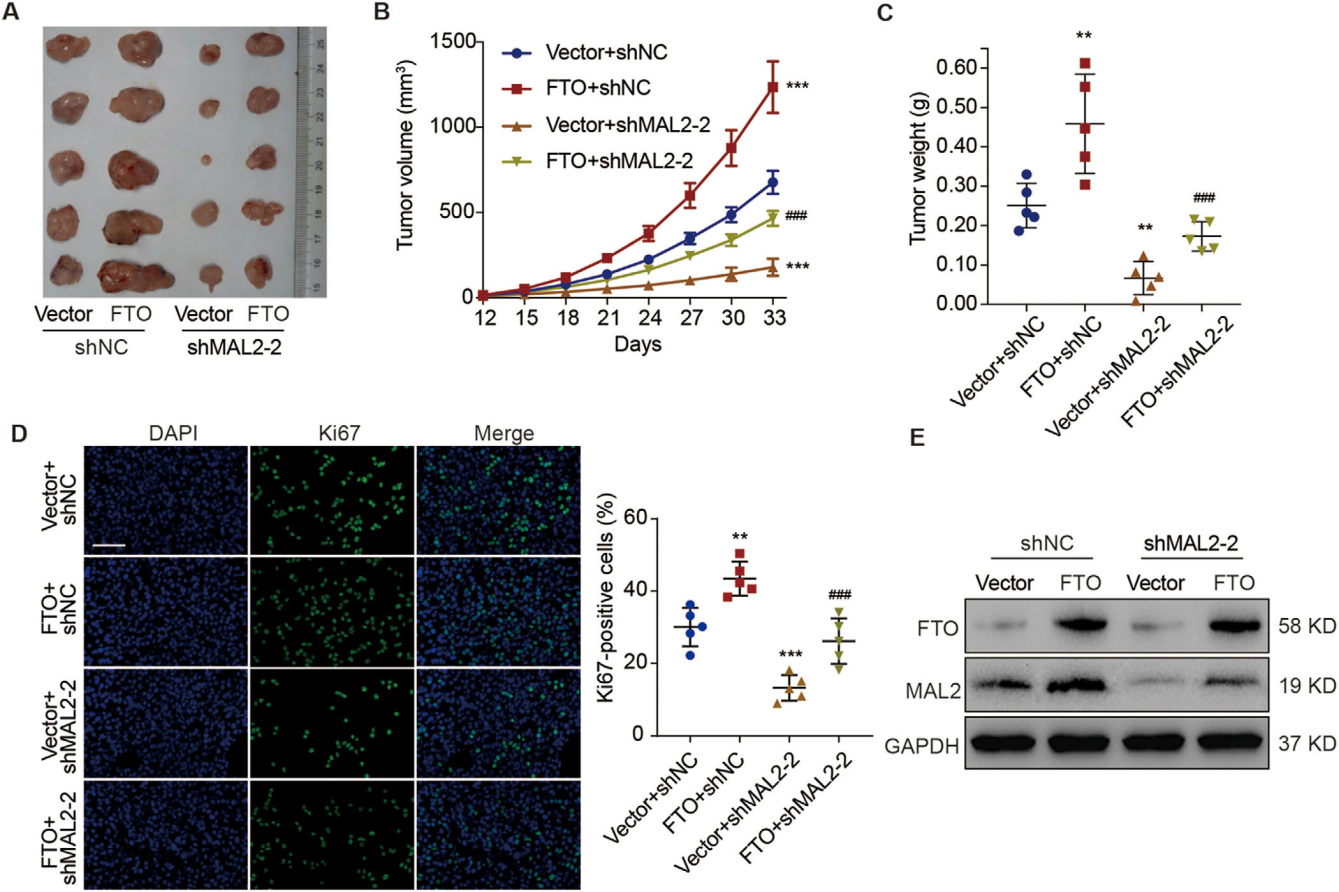
Role of MAL2 in FTO‐mediated tumor growth in bladder cancer. (A) Representative photographs of tumors in mice (n = 5 per group) injected with 5637 cells stably transduced with the indicated constructs at day 33. (B) Tumor volumes of the xenografts. (C) Tumor weights at day 33. (D) Ki‐67 immunofluorescence staining of the xenograft tumors. Scale bar: 100 μm. (E) The protein levels of FTO and MAL2 in the xenograft tumors. One‐way ANOVA (C, D) and two‐way ANOVA (B) followed by Bonferroni's post hoc test were used to perform the comparisons. **p < 0.01, ***p < 0.001 compared with Vector+shNC. ^###^p < 0.001 compared with FTO+shNC

Based on an in vitro cell assay, we found that both miR384 mimics and MAL2 knockdown significantly (p < 0.001) counteracted the promotive effect of FTO overexpression on cell viability in 5637 cells (Figure 5F). Simultaneously, based on an in vivo xenograft model, we observed that mice injected with MAL2‐silenced 5637 cells developed significantly smaller tumors (Figure 6A) and had a considerable counteraction of the stimulatory effect of FTO overexpression on tumor development, reflected by the volume (Figure 6B), weight (Figure 6C), and cell proliferation (Figure 6D) of tumor. In addition, FTO overexpression in 5637 cells could upregulate the protein expression of MAL2 in xenograft tumors tissues (Figure 6E). These data indicate that FTO promotes MALAT1/miR384/MAL2 axis‐dependent cell proliferation in bladder cancer cells.

### 3.5 FTO, MALAT1, miR‐384, and MAL2 are clinically relevant in bladder cancer patients

**FIGURE 7:**
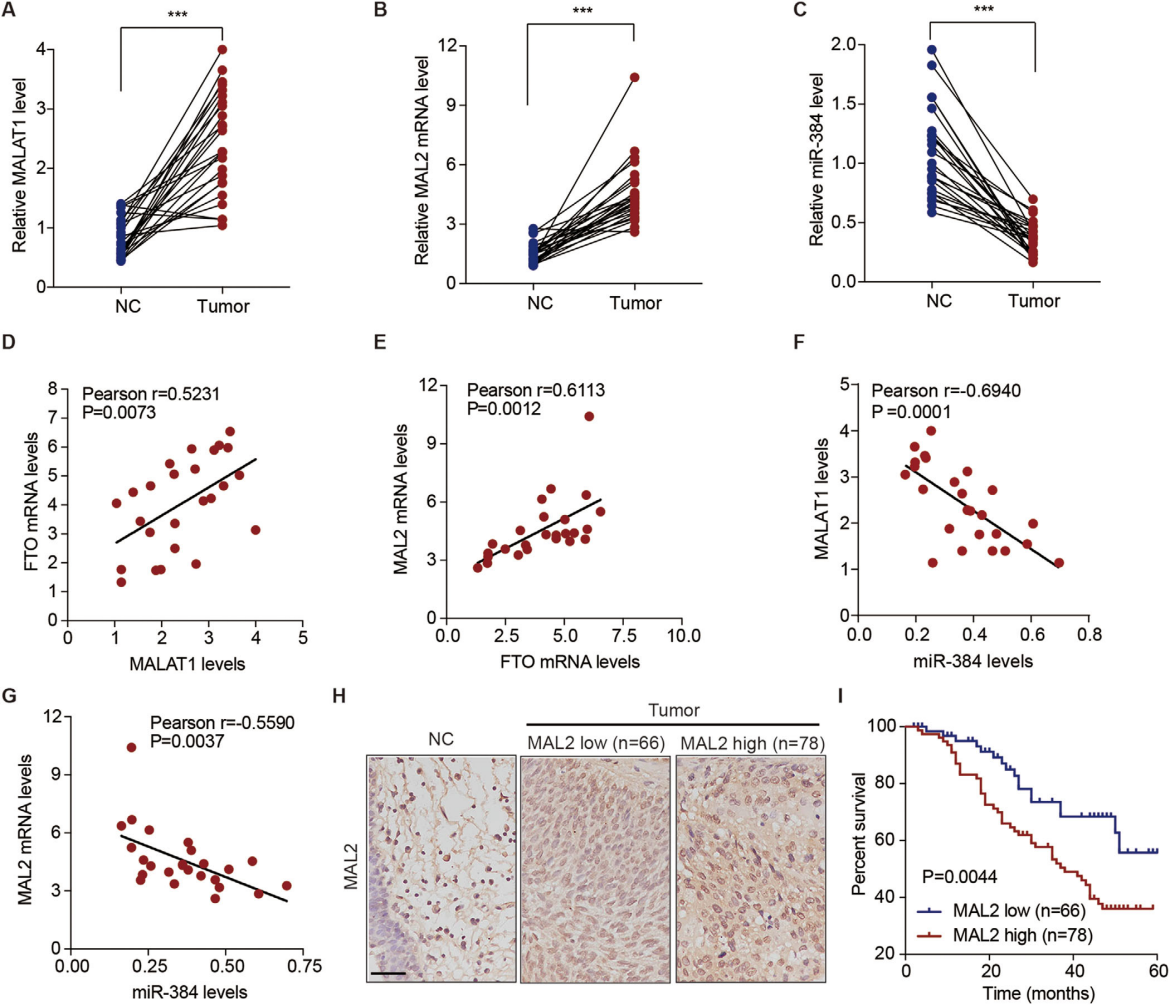
MALAT1, miR‐384, and MAL2 expression in bladder cancer tissues and correlation analyses. The expression levels of MALAT1 (A), MAL2 (B), and miR‐384 (C) in tissues from bladder cancer patients in cohort 1, with comparisons between (n = 25 for each) normal (NC) and tumor paired bladder tissues. (D–G) Pearson's correlation scatterplots of bladder cancer tissues from the patients in cohort 1. (H) The protein expression levels of MAL2 based on the IHC scores of bladder cancer tissue microarrays from the patients in cohort 2, with comparisons among the normal (NC; n = 10), low expression (n = 66), and high expression (n = 78) bladder tissue groups. Scale bar: 100 μm. (I) Kaplan–Meier plots for the overall survival curve of bladder cancer patients, which are based on the MAL2 protein expression levels according to IHC scores from the patients in cohort 2. (A–C) The Mann–Whitney U test was used to perform comparisons between two groups. (I) The log‐rank test was used to perform comparisons among three or more. ***p < 0.001

In the quantification analysis of the FTO, MALAT1, miR‐384, and MAL2 expression in the tissues collected from cohort 1, we determined their clinical relevance. Firstly, the expression levels of MALAT1 (Figure 7A) and MAL2 (Figure 7B) were significantly (p < 0.001) increased, by 2.9‐fold and 3.0‐fold, respectively, in the tumorous tissues of bladder cancer patients, whereas the expression level of miR‐384 (Figure 7C) was significantly (p < 0.001) decreased, by 66.3%, compared with the normal tissues. FTO and MALAT1 expressions were significantly correlated (r = 0.5231, p = 0.0073; Figure 7D). Similarly, FTO and MAL2 expressions were also highly correlated (r = 0.6113, p = 0.0012; Figure 7E). However, a significant negative correlation between MALAT1 and miR‐384 expressions was detected (r = ‐0.6940, p = 0.0001; Figure 7F) and between miR‐384 and MAL2 expressions (r = ‐0.5590, p = 0.0037; Figure 7G). In dividing the tissues from cohort 2, the quantification of MAL2 protein expression scores (Figure 7H), demonstrated that the protein expression of MAL2 was markedly (p = 0.0044) correlated with the survival rate in the bladder cancer patients (Figure 7I). In addition, a significant correlation was found between MAL2 expression and the smoking status (p = 0.0192) and pathologic stage (p = 0.0104) of the bladder cancer patients in cohort 2 and between MAL2 expression and FTO expression (Table 5).

**TABLE 5:** Clinicopathological features of bladder cancer patients and MAL2 expression

	MAL2		
Clinicopathological parameter	Low group, number of patients	High group, number of patients	*p* value
**Age**			0.1501
≤55	35	32	
>55	31	46	
**Sex**			0.3366
Female	28	27	
Male	38	51	
**Tumor size (cm)**			0.2538
≤4	32	40	
>4	44	38	
**Smoke**			0.0192
Yes	26	46	
No	40	32	
**Pathologic stages**			0.0104
pTa‐T1	42	30	
pT2‐T4	24	48	
**FTO level**			0.0071
Low	34	32	
High	23	55	

p < 0.05 represents statistical significance (chi‐square test).

## 4 DISCUSSION

The association between polymorphisms of the FTO gene and the clinical implications of multiple human cancer types has been reported previously.^18^ Despite the fact that Wen et al suggested that FTO is downregulated in bladder urothelial carcinoma,^19^ which was a claim based on mRNA and protein analyses of tissue samples collected from a hospital patient and microarray cohort, we demonstrated that FTO is upregulated in the tumor xenografts obtained from bladder cancer patients. The differences in tissue sample sources, patient conditions, and time of collection might explain these opposing findings. Moreover, FTO protein expression is clinically related to multiple clinicopathological traits of bladder cancer and is highly related to the prognosis in patients, which are consistent with the findings of a research that suggested that FTO expression is connected with the occurrence and prognosis of gastric cancer.^20^ Consequently, we proposed that FTO could be an important molecular biomarker and prognostic indicator of bladder cancer patients.

In the majority of bladder cancer cell, an upregulation of FTO expression at both the mRNA and protein levels was shown. This was true for all of the cell lines except the 5637 cell line. Previous studies have reported differential levels of p‐Akt/total Akt between two subtypes of bladder cancer cell lines: p‐Akt/total Akt was low in basal bladder cancer cell lines (SCABER and 5637) and high in either basal/luminal bladder cancer cell lines (T24 and J82),^21^ and transcription factor SP1 serves as a downstream protein in the PI3K/AKT signaling pathway, binding to the FTO promoter and activating transcription.^22^, ^23^ Therefore, the different levels of the Akt/SP1 signaling axis in bladder cancer cell lines may contribute to different FTO expression levels. Moreover, differences in the subtypes (transitional or squamous cell carcinoma) and grades of differentiation, which further the degree of malignancy, in bladder cancer cell lines also may be linked to different FTO expression levels. Hence, future research on the expression of FTO in different bladder cancer cell lines should be performed. On the other hand, FTO was found to be able to stimulate cell viability and facilitate tumor growth in bladder cancer cells. In fact, an oncogenic role of FTO has been suggested by multiple studies, in which FTO was reported to positively influence proliferation and survival of tumor cells but inhibit cell death and differentiation in various types of human cancer cells.^20^, ^24^, ^25^ The current study only focuses on the functional roles of FTO in bladder cancer by analyzing cellular viability *in vitro and tumor* development in vivo. Hence, future research on its functions in other hallmark characteristics of bladder cancer should be performed. For example, FTO might also participate in the restriction of programmed cell death or suppression of cell differentiation, cellular metabolic reprogramming, and even chemoresistance.^5^, ^15^

The main functions of FTO in tumorigenesis are attributed to its characteristic of being an m^6^A demethylase.^26^ The m^6^A modification on mRNA can modulate the expression of cancer‐related genes at the post‐transcriptional level by either enhancing the stability of mRNA or by increasing the translation efficiency of mRNA through recognizing m^6^A reader proteins.^26^, ^27^ In this study, we detected that FTO can facilitate suppression of the m^6^A modification of MALAT1 5′‐end, thus enhancing the half‐life and expression of MALAT1 mRNA. Moreover, silencing of the m^6^A reader protein YTHDF2 enhanced the stability and activity of MALAT1 mRNA. These findings indicate that FTO can regulate MALAT1 through m^6^A demethylation to compromise its mRNA stability and downregulate its cellular expression. However, MALAT1, as one of the long noncoding RNAs that epigenetically and post‐transcriptionally regulates gene expression, has been associated with the tumorigenesis of most cancer types, including bladder cancer.^28^ In the present study, we also observed that MALAT1 knockdown inhibits cell viability in bladder cancer cells, whereas FTO overexpression compromised this inhibitory effect, which demonstrates that the regulatory role of FTO in bladder cancer cell viability is based on its function in MALAT1 demethylation.

Furthermore, the interaction between MALAT1 and miR‐384 was identified in this study, and both of these genes participate in regulating bladder cancer cell viability. In fact, the involvement of miRNA in regulating MALAT1 expression has been documented in the literature, in which several miRNAs target and bind MALAT1 to induce its degradation.^29^ Meanwhile, it is suggested that miR‐384 inhibits the tumorigenesis of renal cancer through regulating cyclin‐dependent kinase 6,^30^ which supports our finding that miR‐384 suppresses bladder cancer cell viability. Moreover, in accordance with a previous study that revealed the inhibitory effect of MALAT1 on glioma tumorigenicity via regulating miR‐384,^31^ we also found that MALAT1 and miR‐384 interact with and modulate the expression level of each other, which further regulates the viability of bladder cancer cells.

Similarly, we also detected the miR‐384‐MAL2 interaction. Previously, it was revealed that microRNA can bind to MAL2 3′‐UTR and suppress its expression.^32^ Correspondingly, miR‐384 downregulated the MAL2 level in this study as well. The novel family member of MAL proteolipid, MAL2, is known to bind with tumor protein D52, thus participating in regulating the tumorigenicity of multiple human cancer types.^33^, ^34^, ^35^ Accordingly, we found that MAL2 silencing could reduce the viability of bladder cancer cells, indicating that it has an oncogenic role in bladder carcinogenesis. In addition, MAL2 was also highly expressed in bladder tumor cells, and its expression associated strongly with the survival rate and clinicopathological traits of bladder cancer. These findings reveal the potential of MAL2 for serving as a prognostic biomarker for bladder cancer, which is supported by the findings from previous studies on the association between MAL2 and prognostic conditions of breast, colorectal, and pancreatic cancers.^34^, ^36^, ^37^

In previous studies, FTO and microRNA genes were demonstrated to be capable of interacting with and affect each other in adipose and tumor tissues, thus regulating adipogenesis and tumorigenesis.^38^, ^39^ In the current study, we illustrated that FTO could suppress the transcript level of miR‐384, and it could induce subsequent upregulation of MAL2 expression as well. Considering the abovementioned findings related to FTO and MALAT1, as well as the clinical relevance of FTO, MALAT1, miR‐384, and MAL2, we speculate that FTO directly catalyzes MALAT1 demethylation, interferes with the interplays among MALAT1, miR‐384, and MAL2, and, consequently, indirectly regulates the cellular levels of miR‐384 and MAL2.^40^, ^41^, ^42^, ^43^, ^44^ Although we have demonstrated that FTO can influence the cell viability and tumorigenicity of bladder cancer through regulating the MALAT1/miR‐384/MAL2 axis, the detailed molecular mechanisms of the interactions among these four factors, which are correlated with bladder cancer and clinically relevant with each other, warrant further investigation.

In summary, the FTO gene is identified as an oncogenic gene involved in the tumorigenesis of bladder cancer. A high expression of FTO is detected in tumor tissue and is clinically related to the pathological traits of bladder cancer, suggesting that FTO is a potential biomarker for either the diagnosis or clinical prediction of bladder cancer. Moreover, FTO promotes the tumorigenicity of bladder cancer, specifically stimulating tumor growth in vivo and cellular viability in vitro. We demonstrate that the mechanism underlying the oncogenic effect of FTO is based on its capability of MALAT1 demethylation and the regulatory functions on the MALAT/miR‐384/MAL2 axis, which are three of the crucial molecular biomarkers clinically relevant to bladder cancer. Therefore, we have systematically illustrated the oncogenic role of FTO in bladder cancer and its molecular mechanism in the process. This study provides a solid foundation for further validation and development of FTO as diagnostic predictor, prognostic marker, or even therapeutic target for bladder cancer.

## CONFLICT OF INTEREST

The authors declare that there is no conflict of interest that could be perceived as prejudicing the impartiality of the research reported.

## AUTHOR CONTRIBUTIONS

Le Tao, Xingyu Mu, and Zhihua Zhou designed studies, analyzed, and wrote the manuscript. Xingyu Mu, Haige Chen, Yuyang Zhao, and Jie Fan conducted the in vitro and in vivo experiments and data analysis. Di Jin, Ruiyun Zhang, and Ming Cao collected the patient samples, follow‐up information, and performed the clinical data analysis. Le Tao and Zhihua Zhou performed the bioinformatics analysis. All the authors read and approved the final manuscript.

## AVAILABILITY OF DATA AND MATERIALS

The data that support the findings of this study are available from the corresponding author upon reasonable request.

## Supporting information

SUPPORTING INFORMATION

SUPPORTING INFORMATION

SUPPORTING INFORMATION
