# Supplementary material for: FTO modifies the m6A level of MALAT and promotes bladder cancer progression
Source: Clin Transl Med. 2021 Feb 1;11(2):e310. doi: 10.1002/ctm2.310 (PMC7851431; doi:10.1002/ctm2.310)
Supplement: Supplementary file 1 — SUPPORTING INFORMATION [file CTM2-11-e310-s001.docx]

**FTO modifies the m6A level of MALAT and promotes bladder cancer progression**

Le Tao^1,^*, Xingyu Mu^2,^*, Haige Chen^1,^*, Di Jin^1^, Ruiyun Zhang^1^, Yuyang Zhao^2^, Jie Fan^2^, Ming Cao^1^, Zhihua Zhou^3^

^1^Department of Urology, Renji Hospital, School of Medicine, Shanghai Jiaotong University, Shanghai 200080, China

^2^Department of Urology, Shanghai General Hospital, School of Medicine, Shanghai Jiaotong University, Shanghai 200080, China

^3^Department of Urology, Menchao Hepatobiliary Hospital of Fujian Medical University, Fuzhou 350025, China

*Contributed equally to this work

Correspondence to: Zhihua Zhou, Department of Urology, Menchao Hepatobiliary Hospital of Fujian Medical University, 312 Xihong Road, Gulou District, Fuzhou 350025, China. Tel: +86-13370250929, Fax: +86-059-183798317 E-mail: zhihuazhou_fjmu@outlook.com

**Running title:** FTO/MALAT/miR-384/MAL2 axis in bladder cancer

**FUNDING**

This work was supported by the National Natural Science Foundation of China (81702502, 81902594 and 81902562) and Shanghai Municipal Commission of Health Planning (201740024).

**
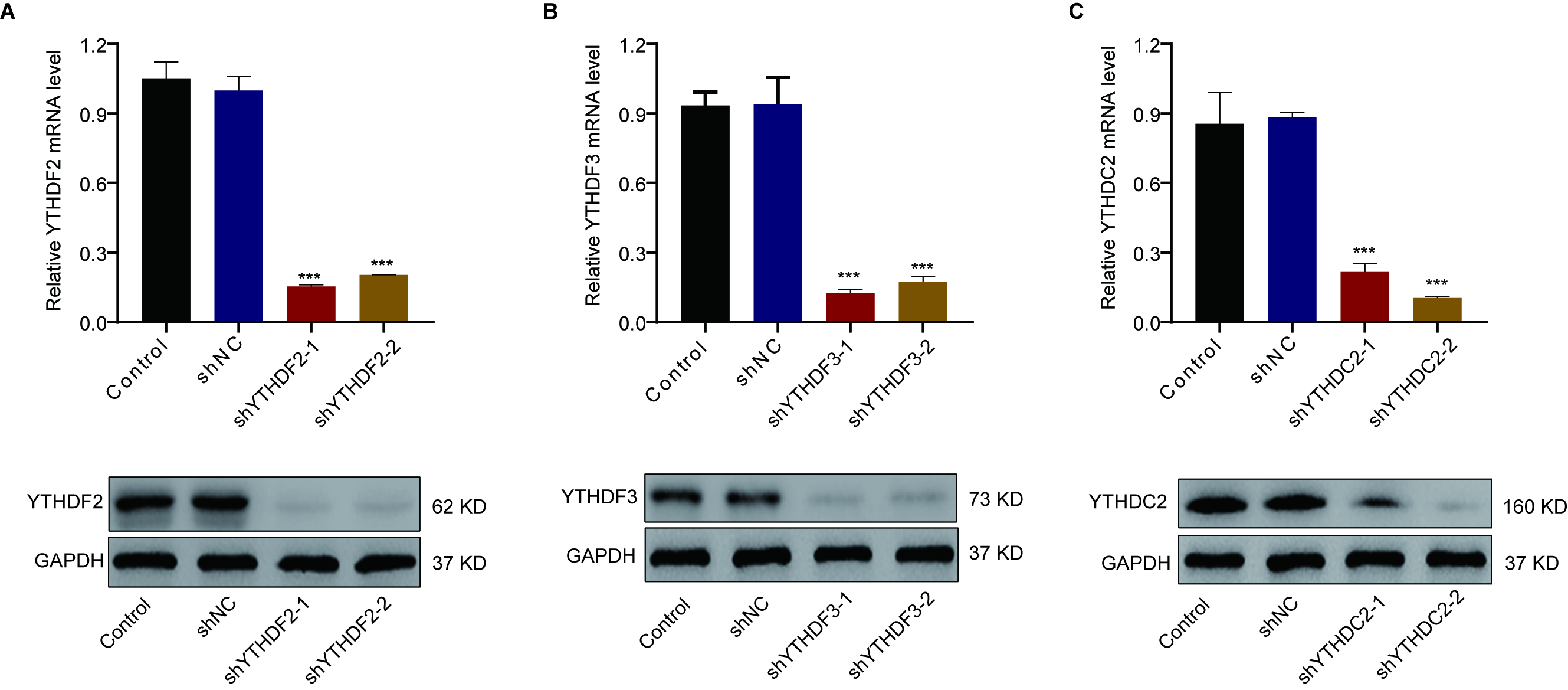
**

**Figure S1. YTHDF2, YTHDF3 and YTHDC2 knockdown in bladder cancer cells.** The 253J cells were transduced with YTHDF2, YTHDF3, or YTHDC2 shRNA vectors. The mRNA and protein levels of YTHDF2 (A), YTHDF3 (B), and YTHDC2 (C) were measured by real time PCR and western blot, respectively. One-way ANOVA followed by Bonferroni’s post hoc test was used for comparisons. ****P*<0.001 compared with shNC.
